# Surgical removal of submacular perfluorocarbon liquid with a 38-gauge flexible cannula combined with internal limiting membrane peeling and intravitreal air tamponade: a case series

**DOI:** 10.1186/s12886-018-0798-y

**Published:** 2018-06-04

**Authors:** Zhongjing Lin, Yanwei Chen, Sha Gao, Yisheng Zhong, Xi Shen

**Affiliations:** 1 Department of Ophthalmology, Ruijin Hospital, Affiliated Shanghai Jiaotong University School of Medicine, 197 Ruijin Er Road, Shanghai, 200025 China; 2 Department of Ophthalmology, Ruijin Hospital North, Affiliated Shanghai Jiaotong University School of Medicine, 999 Xiwang Road, Shanghai, 201801 China

**Keywords:** Perfluorocarbon liquid, Submacular, Internal limiting membrane peeling

## Abstract

**Background:**

To report a case series in which a modified technique was used to remove retained submacular perfluorocarbon liquid (PFCL) secondary to vitreoretinal surgery for rhegmatogenous retinal detachment.

**Case presentation:**

Four patients who had undergone pars plana vitrectomy for rhegmatogenous retinal detachment were further treated with surgical intervention because of retained submacular PFCL. With a three-port pars plana approach, after the internal limiting membrane peeling with indocyanine green staining, a 38-gauge flexible cannula was used to aspirate the submacular perfluorocarbon bubble, followed by fluid-air exchange and air injection into vitreous cavity. Submacular perfluorocarbon liquid was removed successfully and visual acuity had an improvement in all cases.

**Conclusion:**

The surgical removal of retained submacular PFCL using a 38-gauge flexible cannula combined with internal limiting membrane peeling and intravitreal air tamponade may provide anatomical and visual satisfactory outcomes.

**Electronic supplementary material:**

The online version of this article (10.1186/s12886-018-0798-y) contains supplementary material, which is available to authorized users.

## Background

Perfluorocarbon liquid (PFCL) is now in wide and critical use in vitreoretinal surgery. The presence of submacular PFCL is a significant surgery-associated complication and can lead to functional visual loss, central scotoma and irreversible retinal structural damage [1]. Removal of submacular PFCL is generally recommended. However, there is no expert consensus or reference for the standard surgical treatment. Here, we report four cases of surgical removal of retained submacular PFCL bubbles using a 38-gauge flexible cannula combined with internal limiting membrane (ILM) peeling and intravitreal air tamponade.

## Case presentation

### Case 1

**Fig. 1:**
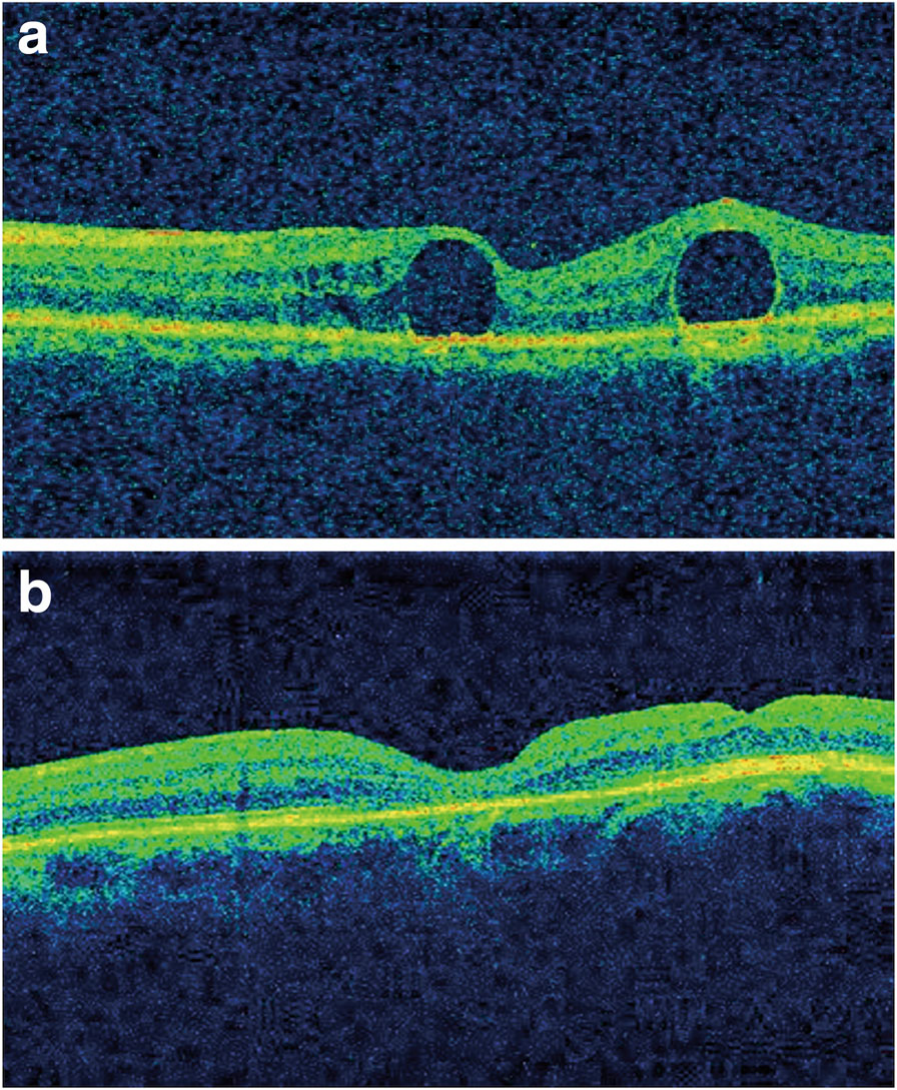
Multimodal imaging of the affected eye. **a** Preoperative OCT showing two submacular PFCL bubbles. **b** Postoperative OCT showing restoration of the foveal contour

A 71-year-old male, presented with rhegmatogenous retinal detachment in the right eye, underwent 23-gauge vitrectomy, perfluoropropane (C_3_F_8_) gas tamponade, as well as phacoemulsification with intraocular lens implantation. Three weeks later, when the patient visited our clinic for the first time postoperatively, 2 bubbles of submacular PFCL were detected on optical coherence tomography (OCT) scans (Fig. 1a). The patient was further treated with a three-port pars plana vitrectomy (PPV). At the time of surgery, after the ILM peeling with indocyanine green (ICG) staining, direct aspirations of PFCL were performed using a 38-gauge needle placed on the top of the bubbles. This was followed by fluid-air exchange and air injection into vitreous cavity. One month later, visual acuity improved from 20/1000 to 20/60, and OCT scans showed a relatively well-preserved macular appearance (Fig. 1b).

### Case 2

**Fig. 2:**
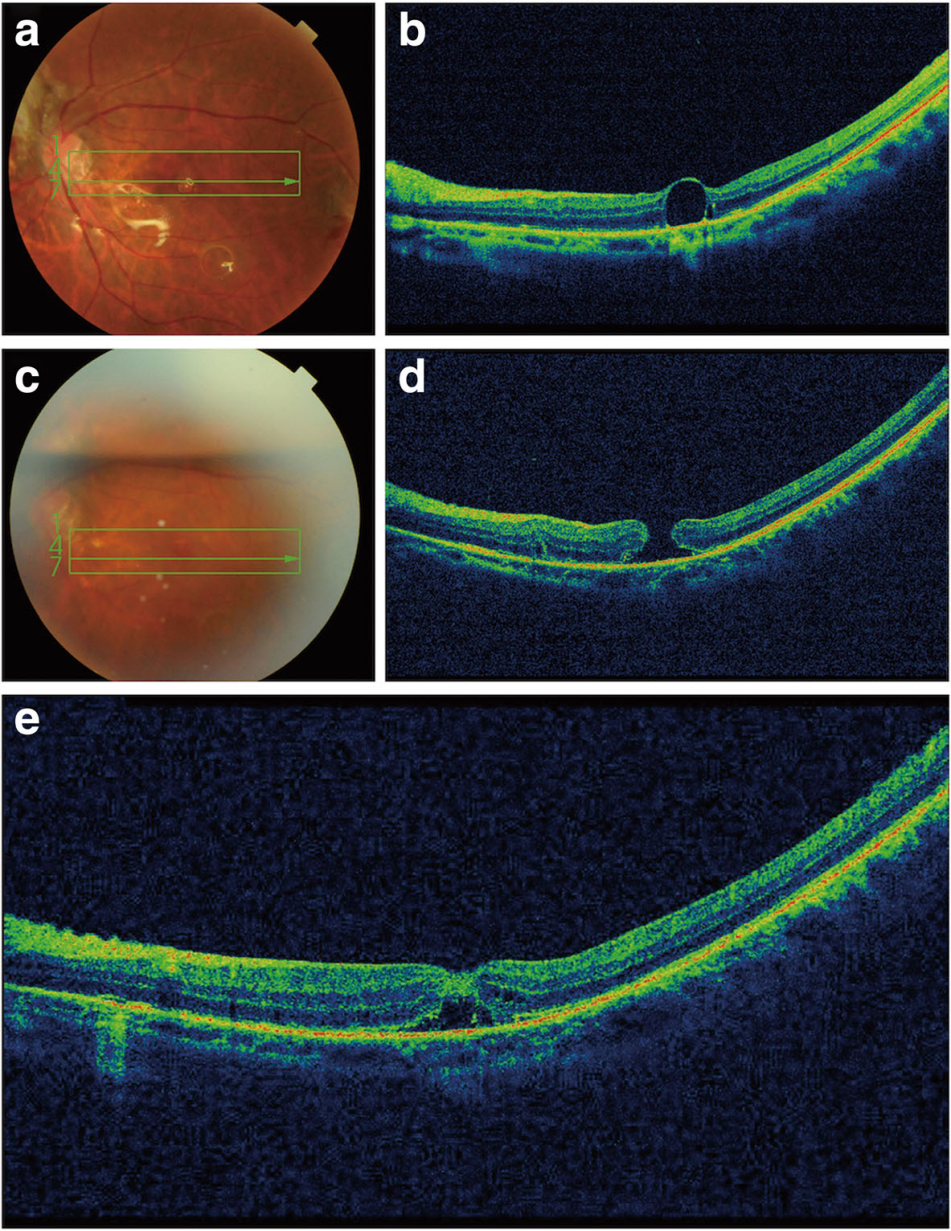
Multimodal imaging of the affected eye. **a** Fundus photograph showing a subfoveal PFCL bubble after the first surgery. **b** OCT showing a small PFCL droplet under the retina after the first surgery. **c** Postoperative fundus photograph showing a macular hole. **d** Postoperative OCT shows a full-thickness macular hole. **e** OCT showing a flat open macular hole

A 52-year-old male with visual loss in the left eye was referred to our clinic. Fundus examinations suggested retinal detachment. The patient underwent 23-gauge vitreoretinal surgery, involving PFCL injection, laser photocoagulation around the retinal tear and peripheral retinal cryopexy for lattice degeneration. Silicone oil was injected as an intraocular tamponade after fluid-air exchange. Four weeks later, at the time of the first follow-up clinic visit, subfoveal PFCL retention was observed by funduscopy and OCT (Fig. 2a and b), and visual acuity of the left eye was limited to 20/1000. The patient underwent silicone oil removal, PPV, ILM peeling, PFCL aspiration and air injection. Two weeks postoperatively, the patient complained about a central scotoma and visual deformation of the affected eye. After careful examinations, the PFCL droplet disappeared but a full-thickness macular hole was noticed (Fig. 2c and d). Additional vitrectomy with 14% C_3_F_8_ injection was performed to enhance macular hole closure. Six months after the last surgery, OCT still revealed a flat open macular hole (Fig. 2e). Visual acuity was stable at 20/125 without central scotoma.

### Case 3

**Fig. 3:**
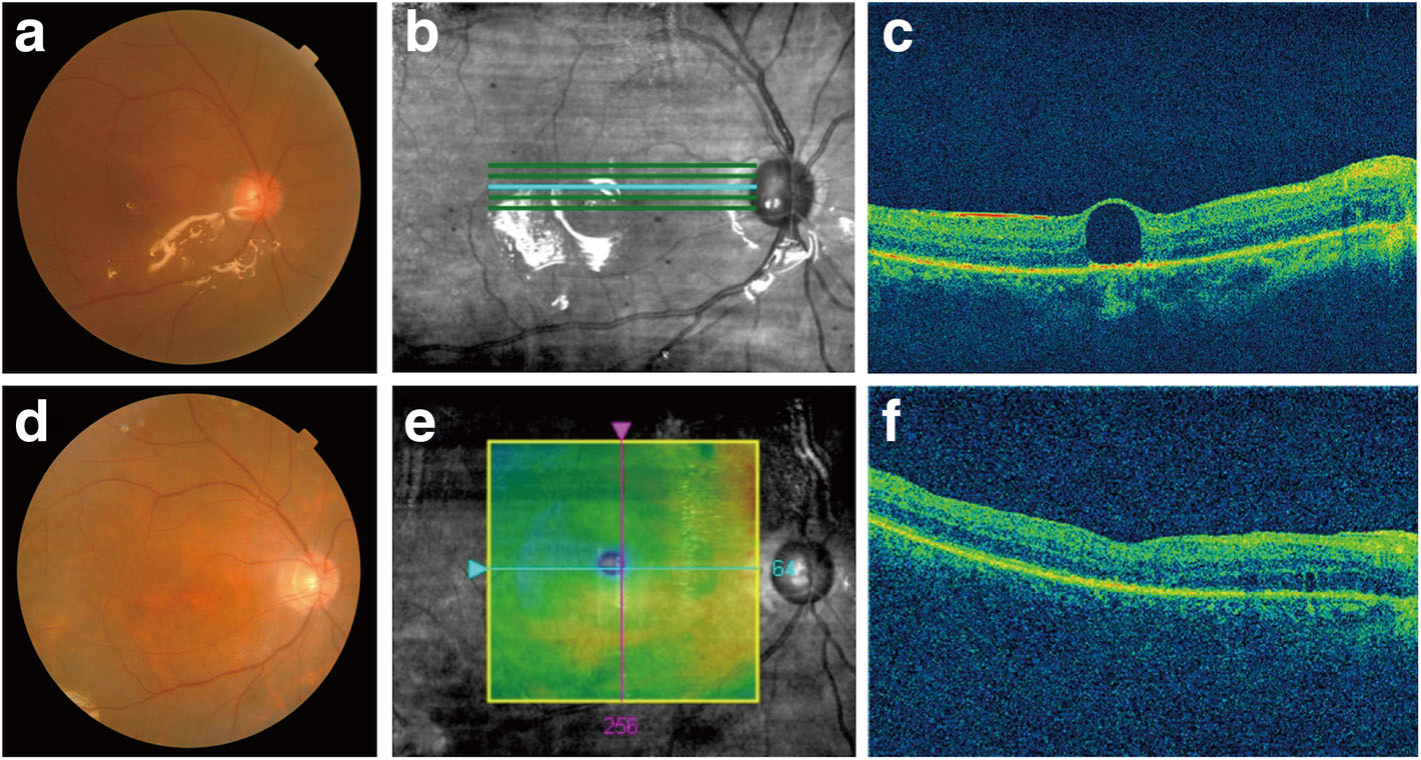
Multimodal imaging of the affected eye. **a** Preoperative fundus photograph showing a small PFCL bubble beneath the fovea. **b** Preoperative images showing the position of OCT scans. **c** Preoperative OCT showing a subfoveal PFCL droplet. **d** Postoperative fundus photograph showing a normal foveal contour. **e** Postoperative images showing the position of OCT scans. **f** Postoperative OCT showing a normal foveal contour

A 42-year-old male complained of impaired vision in the right eye for 5 days and was diagnosed as rhegmatogenous retinal detachment. He had received 23-gauge vitreoretinal surgery with the use of PFCL and silicone oil tamponade. The left eye was normal. Visual acuity improved from counting fingers close to face to 20/1000 after 2 weeks. Although retina was well attached, a 300-μm bleb of subfoveal PFCL was noted (Fig. 3a-c). The patient accepted the surgical option after discussing the possible consequences and outcomes for the management of submacular PFCL. Immediately, the patient underwent PPV, ILM peeling, PFCL aspiration and intravitreal air tamponade. Two weeks postoperatively, visual acuity had improved to 20/100. Funduscopy and OCT revealed a normal foveal contour without edema or subretinal fluid (Fig. 3d-f).

### Case 4

**Fig. 4:**
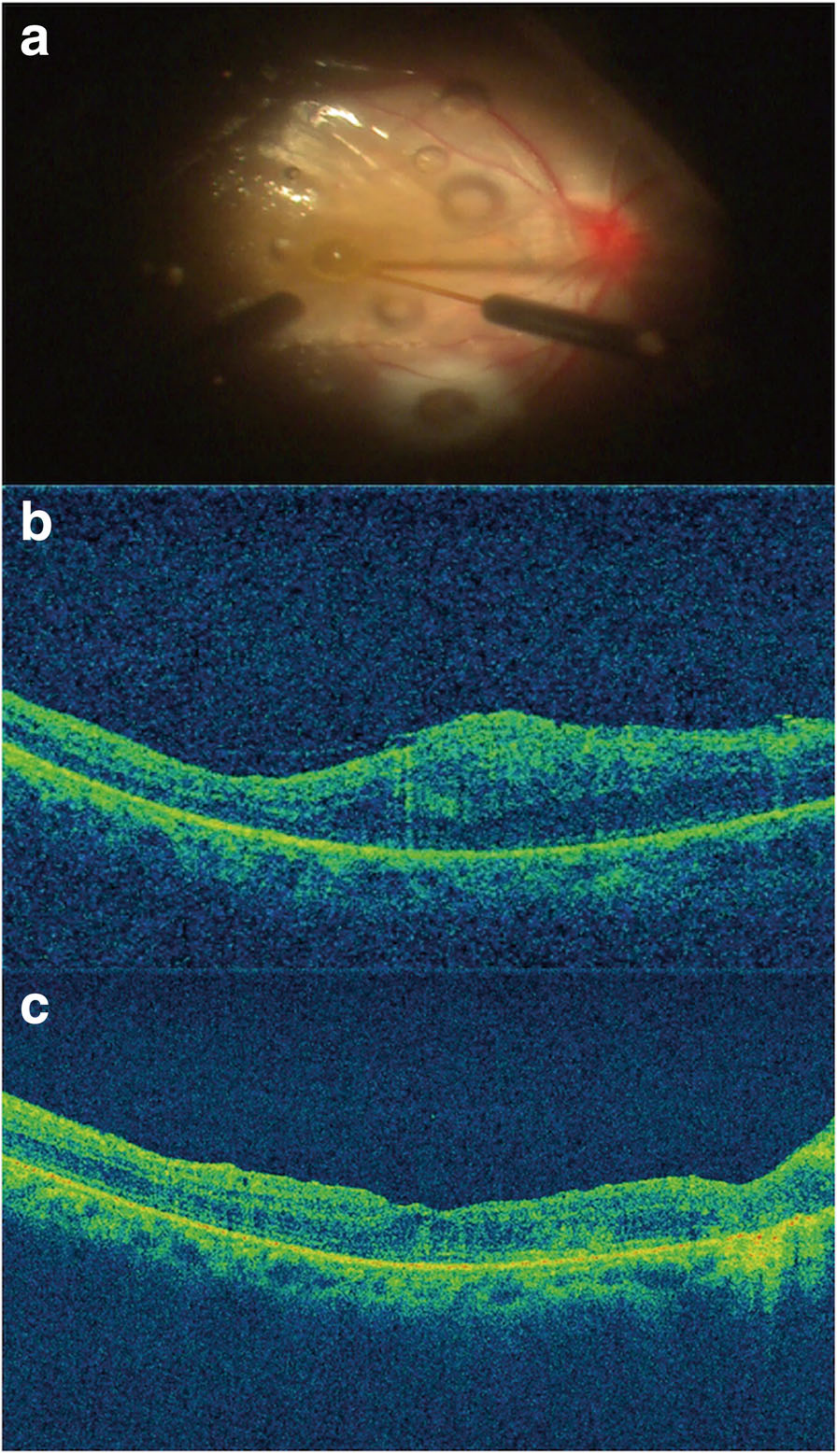
Multimodal imaging of the affected eye. **a** Intraopeative fundus photograph showing the subretinal PFCL. **b** OCT showing the presence of epiretinal membrane and significant macular thickening after the first surgery. **c** OCT showing a normal appearance without signs of edema after the last surgery

A 44-year-old female was referred to our institution with retinal detachment. The patient underwent 23-gauge pars plana vitrectomy, accompanied by intravitreal PFCL injection, retinal laser treatment, and fluid-air exchange, followed by silicone oil tamponade. Prior to the completion of the surgery, submacular PFCL in the juxta foveal location was noticed (Fig. 4a). A 38-gauge needle was inserted into the bubble, and active suction was used to remove the PFCL. Intraoperatively, no significant changes were observed in the macular region beneath the PFCL after its removal. At 1 month postoperatively, the patient complained of metamorphopsia. OCT revealed the presence of epiretinal membrane and significant macular thickening (Fig. 4b), and visual acuity was 20/100. After a thorough discussion, the patient agreed to undergo the surgical intervention. After removing the silicon oil, we performed ILM peeling and intravitreal air tamponade. Two months postoperatively, visual acuity had improved to 20/50, and the macular regained a normal appearance without signs of edema (Fig. 4c).

## Discussion

Subretinal PFCL is a relatively rare but serious complication secondary to surgical repair of rhegmatogenous retinal detachment. Temporary exposure to PFCL has been reported to be clinically safe, however, retained PFCL has the potential toxic effects on various structures of the retina [2]. Actually, it is optimal to notice the PFCL bubbles intraoperatively and remove subretinal PFCL immediately. However, subretinal retention of PFCL cannot be detected in 0.9 to 11.1% of cases until follow-up visits [3].

During vitreoretinal surgery, especially for macular-off retinal detachment or giant retinal tears, unnoticed small droplets could migrate into the subretinal space via retinal breaks during PFCL injection. The high jet stream of the injection may induce turbulence in the vitreous cavity, causing disruption of the PFCL surface tension, thus increasing the possibility for PFCL bubbles to migrate towards the subretinal space. If the end of the injection needle is not completely submerged into the growing bubble, subretinal PFCL droplets may also occur. Once PFCL was displayed in the subretinal space, long-term retention may induce progressive retinal pigment epithelial and photoreceptor damage, resulting in irreversible visual impairment [4]. Visual prognosis of residual PFCL depends on the location, size and exposure time. Since the removal of the retained subretinal PFCL is challenging and may cause significant damages, prompt intervention appears to be recommended particularly in the submacular location.

In our cases, a modified minimally traumatic technique was used for direct aspiration of the retained PFCL bubbles. A standard three-port pars plana vitrectomy was performed in all patients. The ILM of the retina was stained with the use of ICG and then peeled to the vascular arcades. Subsequently, a 38-gauge flexible cannula was used to carefully aspirate the PFCL droplets, with the tip in a substantially perpendicular position. Additional file 1: Video S1 shows the procedure in more detail. Postoperatively, the patients achieved both an anatomic success and an improvement in visual acuity. Despite in one case, the patient developed a macular hole which was successfully resolved with another surgical intervention, this method should be considered as alternative for the treatment of retained submacular PFCL. Further studies with larger patient population and longer follow-up are warranted to verify and extend our findings. Moreover, it is worth noting that, in case 4, direct ILM peeling was not attempted at the beginning as silicon oil had already been injected into the vitreous cavity. We observed the development of epiretinal membrane postoperatively, which could be due to the perifoveal retinal puncture and a predisposition in young patients [5]. More specifically, PFLC aspiration could make an open break, facilitating the access of retinal pigment epithelial cells or fibroblast-like cells to the epiretinal surface and to produce collagen. Further histopathologic studies on epiretinal membrane are needed to better demonstrate this problem.


**Additional file 1: Video S1.** Shows the surgical procedure in more detail. (MP4 93160 kb)


There are several issues that deserve further discussions. First, there is no consensus about the optimal timing for submacular PFCL removal. But in general, earlier removal seems to be more appropriate to improve visual functions and to avoid further retinal damages. As we all know, the photoreceptor inner segment/outer segment (IS/OS) junction has been found to be an important prognostic factor for visual acuity. In our current cases, we noticed that the appearance of photoreceptor layers seemed to be defective, thus necessitating earlier surgical interventions. If we don’t consider the time periods when submacular PFCL is detected, and the patient is willing to accept the surgical intervention, the procedure is recommended in 2 weeks after the initial surgery. Based on our results and the limited number of previously published case reports [6–8], follow-up results indicated that early surgical removal generally showed a good prognosis. Second, ILM peeling is a technical procedure applied to release tangential traction, thus minimizing the risk of an iatrogenic macular hole. On the other hand, since the inner retina seems to be more rigid when the ILM exists, ILM peeling might weaken the force acting to confine PFCL in the subretinal space and increase retinal flexibility. Cillà et al. [9] also affirmed the significance of ILM peeling and further demonstrated that performing the procedure immediately before or shortly after the PFCL aspiration did not seem to influence anatomic results. Third, different sizes of cannulas were used for PFCL aspiration in previous reports, ranging from 36-gauge to the smallest 50-gauge [10–13]. It is challenging to select the most suitable cannula and the choice will largely depend on the size and location of PFCL, as well as the technical considerations of the surgeons. While aspirating the subretinal PFCL, the tip of the cannula should be placed exactly on the top of the bubble in a substantially perpendicular position, so as to minimize the potential damage to retinal functions. Fourth, since the risk of postoperative macular hole could not be excluded, all patients were finally treated with intravitreal air injection and instructed to maintain a face-down position for at least 3 days. Intravitreal gas tamponade is supposed to be beneficial for macular hole closure after elimination of the tangential force. In addition, it provides a template for glial cell proliferation and migration.

## Conclusion

In conclusion, the surgical removal of subretinal PFCL using a 38-gauge flexible cannula combined with internal limiting membrane peeling and intravitreal air tamponade may provide anatomical and visual satisfactory outcomes.

## Abbreviations

C_3_F_8_: Perfluoropropane
ICG: Indocyanine green
ILM: Internal limiting membrane
OCT: Optical coherence tomography
PFL: Perfluorocarbon liquid
PPV: Pars plana vitrectomy
